# Using AI to Detect Pain through Facial Expressions: A Review

**DOI:** 10.3390/bioengineering10050548

**Published:** 2023-05-02

**Authors:** Gioacchino D. De Sario, Clifton R. Haider, Karla C. Maita, Ricardo A. Torres-Guzman, Omar S. Emam, Francisco R. Avila, John P. Garcia, Sahar Borna, Christopher J. McLeod, Charles J. Bruce, Rickey E. Carter, Antonio J. Forte

**Affiliations:** 1 Division of Plastic Surgery, Mayo Clinic, Jacksonville, FL 32224, USA; 2 Department of Physiology and Biomedical Engineering, Mayo Clinic, Rochester, MN 55902, USA; 3 Division of AI in Health Sciences, University of Louisville, Louisville, KY 40292, USA; 4 Department of Cardiovascular Medicine, Mayo Clinic, Jacksonville, FL 32224, USA; 5 Department of Health Sciences Research, Mayo Clinic, Jacksonville, FL 32224, USA

**Keywords:** artificial intelligence, AI, machine learning, pain, facial expression

## Abstract

Pain assessment is a complex task largely dependent on the patient’s self-report. Artificial intelligence (AI) has emerged as a promising tool for automating and objectifying pain assessment through the identification of pain-related facial expressions. However, the capabilities and potential of AI in clinical settings are still largely unknown to many medical professionals. In this literature review, we present a conceptual understanding of the application of AI to detect pain through facial expressions. We provide an overview of the current state of the art as well as the technical foundations of AI/ML techniques used in pain detection. We highlight the ethical challenges and the limitations associated with the use of AI in pain detection, such as the scarcity of databases, confounding factors, and medical conditions that affect the shape and mobility of the face. The review also highlights the potential impact of AI on pain assessment in clinical practice and lays the groundwork for further study in this area.

## 1. Introduction

Pain is an unpleasant subjective experience caused by actual or potential tissue damage associated with complex neurological and psychosocial components [1,2]. Self-reporting is the primary method of assessing pain, as it is highly individualized and dependent on the individual’s perception [3,4].

The medical literature provides several pain scoring systems for pain assessment, including the 100 mm visual analog scale (VAS), the numeric rating scale (NRS), and the color analog scale [5,6,7]. Studies have shown that the VAS is a highly reliable and valid measure of pain and is the most responsive to treatment effects based on substantial evidence [8].

Despite its value, the VAS is beset by several shortcomings. For instance, it is not feasible to employ it in situations where the individuals are either unconscious, cognitively impaired, or unable to articulate themselves verbally [9].

Observational scales have been developed and validated for use in different clinical settings and with specific patient populations to address patients’ inability to communicate their pain. These scales, such as the Behavioral Pain Scale, Nociception Coma Scale, and Children’s Revised Impact of Event Scale [10,11,12], offer an alternative method for assessing pain but are limited by the observer’s previous training and ability to interpret the pain responses accurately.

Additionally, studies have found that observer biases can affect the results of these scales [13,14,15]. Therefore, there is a need for a genuinely objective pain assessment method that is also time-sensitive to detect changes in the patient’s pain experience.

Artificial intelligence (AI) has the potential to transform the healthcare system by making the analysis of facial expressions during pain more efficient and lessening the workload of human professionals. In particular, AI can automate feature extraction and perform repetitive and time-consuming tasks requiring much human effort by utilizing machine learning (ML) algorithms and data analysis techniques; this may result in better patient outcomes, better use of resources, and lower operating costs [16,17].

The potential of AI in medical imaging analysis has recently come to light in recent studies. Large datasets of medical images, including computed tomography scans and X-rays, can be used to train AI algorithms to recognize abnormalities that point to the presence of a disease. These algorithms have been shown to perform better than human radiologists in some diagnostic tasks [18,19], highlighting the potential of AI to increase the accuracy and efficiency of medical imaging analysis.

### Research Question and Objectives

This study aims to explore the current state of AI-assisted pain detection using facial expressions. The specific objectives of this study are as follows:

1. Summarize the current state of research in this field.
2. Identify and discuss the potential implications and challenges of deploying this technology in the healthcare system.
3. Determine research gaps and propose areas for future work.

## 2. Materials and Methods

For the literature review, we conducted a search on 23 January 2023 using keywords in 4 databases: PubMed, EMBASE/MEDLINE, Google Scholar, Cumulative Index of Nursing and Allied Health Literature (CINAHL), and Web of Science, to identify relevant literature and evidence on the use of AI and ML to detect pain through facial expressions. Posteriorly, we conducted a narrative synthesis to provide a comprehensive overview of the current state of the art, the potential for clinical use, challenges, limitations, ethical concerns, and knowledge gaps for future research.

## 3. Objective Pain Measurement and AI

There has been considerable research on pain responses to develop a more “objective” way of assessing pain. Pain responses include changes in physiological parameters such as galvanic skin response, pupil reflexes, blood pressure, heart rate variability, and hormonal and biochemical markers [20,21,22,23,24]. Additionally, behavioral pain responses can be verbal, such as describing or vocalizing pain, and nonverbal, such as withdrawal behavior, body posture, and facial expressions [25,26,27].

Most attempts to recognize facial expressions have focused on the identification of action units (AUs), defined in the Facial Action Coding System (FACS) [28]. Numerous AUs in the FACS have been linked to pain. However, according to Prkachin (1992), the ones that convey the most information regarding pain are brow lowering, eye closure, orbit tightening, and levator muscle contraction [29]. These four “core” factors also contribute to the majority of the heterogeneity in pain expression [30].

However, the facial indicators of pain that have been validated in the past are impractical for clinical settings due to their reliance on highly skilled observers to label facial AUs, a time-consuming task unsuitable for real-time pain assessment [31,32]. Nevertheless, facial expressions are advantageous in AI/ML because they can provide relevant data in each video frame and changes over time, and computer vision systems could perform this operation automatically through the training of a classifier to recognize the facial expressions connected to pain [33].

### Models Using AI/ML for Pain Detection through Facial Expressions

The first step for the automated detection of pain tasks is to develop a pre-trained ML system. For supervised ML models, this step involves training with large datasets labeled with the correct output, processed by algorithms and mathematical models to recognize patterns associated with the output. Afterward, the inferential phase is started, where the ML model is loaded with new data to generate categorizations. Typically, a camera records video data of a subject’s face. The facial features are then extracted from the video data using computer vision techniques to identify pain-related patterns. These facial features found in frames or video sequences are later processed by the pre-trained ML models, providing their estimation of the subject’s pain experience [34,35].

**Figure 1:**
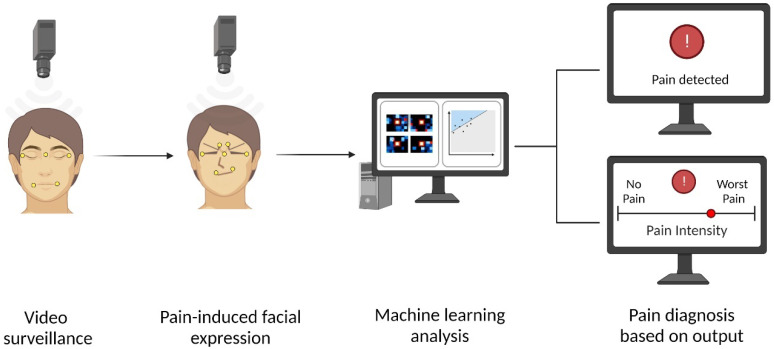
Automated pain detection using AI. This image depicts video surveillance being used to capture facial expressions associated with pain, which are then analyzed by a computer system using machine learning to provide an accurate output of pain detection or intensity estimation. Created with BioRender.com (accessed on 14 March 2023).

Figure 1 depicts a standard proposed scenario for detecting pain through video surveillance of patient faces using computer vision and ML techniques.

## 4. Current Evidence of AI-Based Pain Detection through Facial Expressions

Several studies have found promising findings on the precision of AI-based pain detection using facial expressions. Table 1 summarizes the results of 15 experimental studies that used AI/ML to detect pain using facial expressions.

Overall, the studies showed varying levels of accuracy in pain intensity estimation and detection of pain, with some models performing better than others.

The principal outcomes differed among studies. For instance, one study focused only on the detection of pain [35], eight studies only on the estimation of multilevel pain intensity [36,37,39,41,42,44,45,48], and four studied both the detection of pain and the assessment of multilevel pain intensity [40,46,47,49]. Additionally, two studies proposed their automated detection model to differentiate between genuine and faked facial expressions of pain [38,43].

All the presented studies included videos featuring patients’ faces experiencing varied pain levels, including the absence of pain. AI/ML models were trained and tested on these videos to evaluate their performance in detecting pain through facial expressions.

Four studies applied their automated pain detection systems to videos from their recruited patients [36,38,43,49], and eleven used them on at least one public database of pre-recorded patients experiencing pain [35,37,39,40,41,42,44,45,46,47,48].

From the 11 studies using public databases, 7 used only one database [35,39,40,41,42,46,47], while 3 used a second database to validate further their AI/ML model [37,45,48]. The most used was the UNBC-McMaster Shoulder Pain Archive database, utilized in 10 studies; this consisted of videos of 25 subjects with unilateral shoulder injuries whose pain was elicited by passive and active arm movements [35,37,40,41,42,44,45,46,47,48]. Two studies used the MintPAIN database, consisting of videos of 20 participants with induced pain from electrical stimulation.

One study used the BioVid database (part A), involving 87 subjects experiencing induced painful heat stimuli [48]. Lastly, one study used the X-ITE Pain database, consisting of 127 individuals whose pain was caused by heat and electrical stimulation [39].

Of the four studies that recruited patients for AI/ML model assessment, one consisted of 1189 patients undergoing different surgeries in a single healthcare center [36]. In addition, two studies assessed pain induced through cold pressor methods in 26 healthy university students [43] and healthy volunteers [38]. Lastly, one study consisted of 50 children who underwent laparoscopic appendectomies, assessing their baseline and palpation-induced pain during the preoperative stage and 3 days post operation [49].

## 5. Discussion

### 5.1. The Ground Truth for Pain Assessment

In the context of pain recognition, ground truth refers to the labels that are used to train and evaluate pain recognition systems. There are three types of ground truth: self-report, observer assessment, and study design [50]. Self-report scales are widely considered the gold standard for measuring pain intensity [51,52]. Observer assessment can be conducted with subjective or validated systematic observation scales, and despite being advantageous in particular populations unable to report pain, it might have limited accuracy, especially in untrained observers [53,54]. Study design ground truth is based on prior knowledge about the circumstances in which pain is likely to be felt, such as the effects of certain procedures [55].

In the studies presented in Table 1, the ground truth for the pain assessment varied among studies. The validated Prkachin and Solomon Pain Intensity (PSPI) scale was the most frequently used ground truth scale, used in nine studies [35,37,40,41,42,44,45,46,48]. In addition, four studies relied on self-reported pain on different scales [36,39,47,49]. Finally, five studies relied on study design ground truth; of these, three used the intensity of the applied stimuli (i.e., study design ground truth), which was previously calibrated to cause different levels of pain in the participants [37,45,48], and two used circumstantial knowledge of painful stimulation [38,43].

### 5.2. Is PSPI Suitable for Estimating Pain?

Recent advances in automatic pain estimation have focused on recognizing AUs as defined in the FACS [56]. PSPI is a scale based on frame-level ground truth calculated by assessing AUs [30].

However, the main strength of the PSPI score is its simplicity, as it condenses facial expressions into one number, making it easy to analyze with regression and classification algorithms, thereby leading to its wide acceptance as a tool for measuring pain [35].

Some weaknesses of the PSPI scale are that it does not reflect the experienced pain severity in all cases. There may be instances where a person experiencing pain may have a low PSPI score despite the presence of significant pain or vice versa. For example, some observers may underestimate a patient’s pain experience, and some patients, especially those with motor disorders such as Parkinson’s disease, may not exhibit the facial changes assessed in the PSPI scale [57]. Furthermore, it measures the facial expression of pain but does not provide a comprehensive understanding of the experience of pain, which can be influenced by various factors, including psychological and cultural factors [58].

Regarding the validity of the PSPI scale, research has yielded mixed results regarding the correlation between self-reported pain and facial expressions of pain; however, many studies have demonstrated a significant relationship between both [30,59,60,61].

### 5.3. Performance of AI for Pain Detection through Facial Expressions

In the studies presented in Table 1, the reported accuracy for pain detection ranged from 80.9% to 89.59%, while the AUC ranged from 84% to 93.3%. In pain intensity estimation, the accuracy range was between 51.7% and 96%, while the AUC ranged from 65.5% to 93.67%. Finally, the accuracy range was between 85% and 88% for distinguishing between real and faked pain, with an AUC of 91%.

Most research analyzing facial expressions has examined responses to experimental short-term pain anticipated by subjects. However, it could be possible that facial expressions induced by longer-term pain, such as in cancer pain, may differ from acute pain due to a lack of surprises or expectations. Indeed, this variance may explain the difficulty in creating reliable digital tools to evaluate pain through facial expression analysis for clinical use [48,49].

### 5.4. AI/ML Characteristics and Differences

There are variations among studies in the employed feature extraction tools, ML algorithms, data processing techniques, video or image quality, cross-validation techniques, and other factors that can significantly impact the performance of each model [62].

It is notable that studies utilizing varying techniques on the same populations achieved different degrees of performance (Table 1). Furthermore, the feature extraction tools can significantly impact the accuracy of the models, as demonstrated by some studies where different tools were employed using the same classifiers, resulting in varying levels of accuracy [35,41].

Moreover, as shown in Table 1, pain identification and quantification performance varied even within studies that utilized the same video database. 

Although the accuracy variations could be mainly attributed to the feature extraction tools and AI/ML algorithms, further research is necessary to assess the impact of other potential factors.

### 5.5. Combining Facial Expressions with Other Physiological Data as Input

AI/ML has also been applied to assess pain by fusing the information from facial expressions and other physiological and demographic data. Similar to Sikka et al. (2015) [49], other authors also employed their automated pain detection algorithm on children undergoing laparoscopic appendectomies, demonstrating higher accuracy in detecting clinically significant pain when fusing facial expressions and electrodermal activity as input [63]. Furthermore, other studies have demonstrated that combining facial expression data with demographic and bio-physiological features such as electrocardiograms, electromyography, and skin conductivity can increase the accuracy of pain detection [64,65,66,67].

### 5.6. Machine Learning vs. Human Observers for Pain Estimation

In addition to assessing the performance of the automated detection and quantification of pain, five studies compared the accuracy of human observers to their proposed ML model [36,38,43,49].

Two studies specifically assessed the capability of humans to discriminate genuine vs. faked facial expressions of pain. In the study conducted by Bartlett et al. (2014), trained human observers accurately detected pain in 54.6% of the cases [38]. Moreover, Littlewort et al. (2009) tested human observers and achieved accuracy of 49.1% [43]. In both studies, the authors compared trained and tested ML models, which performed better than human observers (see Table 1), even after training.

Two studies evaluated nurses’ capacity to detect pain in postoperative patients. Fontaine et al. (2022) [36] reported on 33 skilled nurses who estimated pain intensity by looking at facial expressions, with 14.9% accuracy and a mean absolute error of 3.04. Their sensitivity and specificity in the detection of pain (NRS ≥ 4/10) was 44,9% and 68,4%, while for severe pain (NRS ≥ 7/10) the values were 17.0% and 41.1%, respectively. However, the study showed that their AI/ML model outperformed nurses in detecting pain and estimating pain levels, as demonstrated in Table 1 [36]. On the other hand, the results of the study conducted by Sikka et al. (2015) [49] showed that AI/ML performed similarly to nurses estimating baseline postoperative pain and performed better in palpation-induced transient pain. Compared to their ML model’s performance (Table 1), the mean AUC achieved by nurses for pain detection was 0.86 and 0.93 for ongoing and transient pain, respectively; for the pain intensity assessment, nurses estimated ongoing and transient pain intensity with a correlation coefficient of r = 0.53 and r = 0.54, respectively [49]. Moreover, results for automated detection were not impacted by demographic differences, suggesting its advantage against human observers as it does not pose the risk of observer bias [49,68,69].

Lastly, Othman et al. (2021) evaluated the performance of human observers in detecting pain categorized into seven classes, which included three intensities each of heat and electrical pain stimuli and a seventh class for no stimulation. The reported accuracy in the seven-class classification of pain was 21.1%, while for the Convolutional Neural Network classifier accuracy was 27.8% [39].

### 5.7. Potential Applications

The application of AI/ML techniques in the detection of pain through facial expressions presents a plethora of potential advantages. Firstly, it can provide objective and accurate measurements of pain intensity, which can be used to provide more accurate diagnoses and treatments. Additionally, it can be helpful for the detection of pain in situations where it is difficult to assess, such as in patients unable to communicate verbally, critically ill patients, and during the perioperative period [36,49,70,71,72,73].

Inadequate pain management after surgery can have serious consequences, including increased morbidity and mortality, longer recovery times, unexpected hospital readmissions, and chronic persistent pain [74]. Overcoming obstacles to effective pain management, including those related to healthcare providers, is crucial for achieving optimal pain relief after surgery. For example, Sikka et al. (2015) and several other authors have determined that healthcare personnel tend to underestimate children’s self-reported pain [49,75,76], which could be translated to a relevant advantage of AI/ML in assisting healthcare personnel in the effective management of postoperative pain.

By utilizing AI/ML technologies, healthcare providers can analyze and interpret patients’ facial expressions that coincide with pain, ultimately enabling them to customize treatments and dosages based on individual needs. Moreover, an objective and continuous method for monitoring postoperative pain intensity would be highly advantageous, potentially enabling reliable and cost-effective evaluation of pain intensity.

The results of some studies suggest that AI/ML performs better than human observers at differentiating genuine vs. faked pain [38,43]. The practical implications of this capability are broad, including the detection of malingering, which has been reported to be important in patients seeking compensation [77,78,79]. Additionally, it could help prevent insurance fraud and unnecessary narcotics prescriptions, reduce healthcare costs, and ultimately improve the quality of care [36].

### 5.8. Confounding Effect

Evidence suggests that facial expressions of pain are sensitive and specific to pain, and that these expressions can be distinguished from facial expressions associated with basic emotions [80,81]. However, some studies have found that ML algorithms are prone to misinterpreting unpleasant disgust as pain in facial expressions [82]. For instance, Barua et al. (2022) tested their predesigned AI/ML algorithm on the Denver Intensity of Spontaneous Facial Action database, which comprised a set of video frames of the facial expressions of spontaneous emotional expressions. They reported that the proposed pain intensity classification model achieved greater than 95% accuracy in pain detection [44]. Although this database was not designed to study actual pain, AUs associated with pain response are identifiable in video frames, allowing them to be coded using the FACS and the corresponding PSPI scores. Hence, it is essential to consider the specific context in which the automated systems will be used to ensure high accuracy and avoid this confounding effect.

### 5.9. Ethical Concerns

Using AI/ML algorithms to detect pain through facial expressions raises ethical concerns that must be addressed. For instance, it is essential to consider the potential for errors and inaccuracies in pain detection models. Relying only on inaccurate models could lead to dangerous or inappropriate decisions, such as misdiagnosis, inappropriate treatment, or even legal actions [83].

For instance, misdiagnosing certain conditions based on inaccurate pain detection models may lead to low-quality or no care, or prompt unnecessary surgery or medication; this could lead to an erosion of trust between patients and healthcare providers, with the potential for significant legal and financial implications [84].

Additionally, concerns are being raised regarding patient privacy and autonomy. For example, patients should provide informed consent beforehand as they may refuse facial analysis [85,86]. Furthermore, algorithms might be trained for particular demographics, further marginalizing already vulnerable groups [87,88].

## 6. Challenges and Limitations

Automatic pain detection is challenging because it is complex, subjective, and subject to a variety of factors, such as an individual’s personality, social context, and past experiences [89].

Despite the promising results of using AI/ML algorithms to detect pain through facial expressions, they face several limitations. For example, the presence of head motion and rotation, part of typical human behavior in real clinical scenarios, can significantly reduce the accuracy of the AI model’s ability to detect AUs [90,91]. Additionally, its utility may be limited by medical conditions affecting facial shape and mobility, such as Parkinson’s, stroke, facial injury, or deformity [92,93,94,95,96].

The scarcity of diverse databases further limits the development of a reliable and widely generalizable system for recognizing pain through facial expressions [97]. Additionally, differences between sex, age, and pain setting require validation across large pools of data, prompting the debate over whether to adopt a universal approach or create tailored models for each target population [97].

The Hawthorne effect can be considered a potential limitation of the included studies, whereby the participants’ awareness of being observed or filmed may have led to changes in their behavior [98].

Additionally, the application of ML is regarded as a “black-box” method of reasoning, making it challenging to communicate the rationale behind classification choices in a way humans can comprehend [99]. This can be a significant issue as healthcare providers need to understand and interpret the reasoning behind an algorithm’s classification decisions in order to make informed decisions about patient care. Therefore, additional research is required to investigate how to improve the clarity and understanding of the reasoning process.

### Limitations of This Review

Most studies concentrated mainly on the technical elements of automated pain identification, with limited exploration of consequences in healthcare as a whole. It is necessary to consider how these innovations may affect patient care and clinical decision making, even if the technical components of this sector are unquestionably crucial. A more comprehensive strategy that considers both technology and healthcare viewpoints might be advantageous for future research.

Although automated pain recognition could be a particularly valuable tool for specific populations limited to self-reported pain, such as individuals with dementia, newborns, patients under anesthesia, and unconscious patients, these groups remained out of the scope of this review.

Given the multiple factors and confounders that could have altered the accuracy of the AI/ML technologies in detecting pain through facial expressions, we could not establish the most dependable and precise methodology. However, we have exhibited the current state of research in automated pain recognition, identifying trends, capabilities, limitations, potential healthcare applications, and knowledge gaps.

## 7. Conclusions

This review confirms that AI/ML technologies have been used to detect pain through facial expressions to demonstrate their potential to assist during clinical practice. Furthermore, the results indicate that AI/ML can accurately detect and quantify pain through facial expressions, outperforming human observers in pain assessment and detecting deceptive facial expressions of pain. Thus, AI/ML could be a helpful tool in providing objective and accurate measurements of pain intensity, enabling clinicians to make more informed decisions regarding the diagnosis and treatment of pain.

However, it would be wise to encourage the sharing of more diverse and complex publicly available data with the appropriate ethical considerations and proper permissions to allow AI experts to develop reliable and robust methods of facial expression analysis for use in clinical practice. Likewise, well-designed randomized control trials are needed to determine the reliability and generalizability of automated pain detection in real clinical scenarios across medical conditions affecting facial shape and mobility.

Further research is needed to expand the capabilities of AI/ML and test its performance in different pain settings, such as those pertaining to chronic pain conditions, to assess its full potential for use in clinical practice. Additionally, patient satisfaction and preferences regarding the usage and acceptance of AI/ML systems should be explored. Finally, ethical considerations around privacy and algorithm biases are complex and must be addressed.

## Figures and Tables

**Table 1:** Summary of studies assessing the use of AI to detect pain through facial expressions.

Author and Date	Population	Pain Setting	Ground Truth	ML Classifiers	Outcomes	Performance
Fontaine et al. (2022) [36]	Adult patients from a single university hospital	Postoperative pain	NRS	CNN	Pain intensity estimation	Estimation of pain intensity Accuracy = 53% Mean error = 2.4 points Detection of pain (NRS ≥ 4/10) Sensitivity = 89.7% Specificity = 61.5% Detection of severe pain (NRS ≥ 7/10) Sensitivity = 77.5% Specificity = 45%
Bargshady et al. (2020) [37]	UNBC-McMaster database MIntPAIN database	UNBC-McMaster database: self-identified shoulder pain MIntPAIN database: electrical-induced pain	UNBC-McMaster database: PSPI MIntPAIN database: stimuli-based pain levels (0–4)	CNN-RNN	Pain intensity estimation UNBC-McMaster: categorized into five levels (PSPI 0, 1, 2–3, 4–5, and ≥6) MINT: categorized into five levels (0–4)	UNBC-McMaster Accuracy = 86% AUC = 90.5% MSE = 0.081 MAE = 0.103 MIntPAIN Accuracy = 92.26% AUC = 93.67% MSE = 0.0245 MAE = 0.0341
Bartlett et al. (2014) [38]	Healthy subjects	Cold pressor-induced pain	Pain stimuli-dependent assessments	SVM	Detection of genuine vs. faked pain	AUC = 0.91% Accuracy = 85%
Othman et al. (2021) [39]	X-ITE Pain Database	Heat-induced and electrical-induced pain	NRS categorized into 4 pain intensities (no pain, low, medium, and severe)	Two-CNN with sample weighting	Pain intensity detection for electrical and thermal stimuli using two groupings of pain levels: none/low/severe and none/moderate/severe	Mean accuracy = 51.7%
Rodriguez et al. (2022) [40]	UNBC-McMaster database	Self-identified shoulder pain	PSPI	CNN-LSTM	Pain detection Estimation of pain intensity, categorized into 6 levels: PSPI 0, 1, 2, 3, 4–5, and ≥6	Pain detection Accuracy = 83.1% AUC = 93.3% Pain intensity estimation MSE = 0.74 MAE = 0.5
Rathee et al. (2015) [41]	UNBC-McMaster database	Self-identified shoulder pain	PSPI	DML combined with SVM	Detection of pain intensity by PSPI score (16 levels)	Accuracy = 96%
Lucey et al. (2011) [35]	UNBC-McMaster database	Self-identified shoulder pain	PSPI	SVM	Pain detection	Accuracy = 80.9% AUC = 84.7%
Bargshady et al. (2020) [42]	UNBC-McMaster database	Self-identified shoulder pain	PSPI	Hybrid CNN-bidirectional LSTM	Estimation of pain intensity, categorized into four levels: PSPI 0, 1, 2–3, and ≥4	Accuracy = 85% † AUC = 88.7% † MSE = 0.21 † MAE = 0.18 † F-measure = 78.2%
Littlewort et al. (2009) [43]	University students	Cold pressor-induced pain	Pain stimuli-dependent assessments	Gaussian SVM	Detection of genuine vs. faked pain	Accuracy = 88%
Barua et al. (2022) [44]	UNBC-McMaster database	Self-identified shoulder pain	PSPI	K-Nearest Neighbor	Estimation of pain intensity, categorized into four levels: PSPI 0, 1, 2–3, and ≥4	Accuracy = 95.57% Average F1 = 95.67%
Bargshady et al. (2020) [45]	UNBC-McMaster database MIntPAIN database	UNBC-McMaster database: self-identified shoulder pain MIntPAIN database: electrical-induced pain	UNBC-McMaster database: PSPI MIntPAIN database: stimuli-based pain levels (0–4)	Temporal Convolutional Network	Estimation of pain intensity UNBC-McMaster: categorized into four levels: PSPI 0, 1, 2–3, and ≥4 MINT: categorized into five levels (0–4)	UNBC-McMaster Accuracy = 94.14% AUC = 91.3% MSE = 0.186 MAE = 0.234 MIntPAIN Accuracy = 89% AUC = 92% MSE = 0.22 MAE = 0.26
Rathee et al. (2016) [46]	UNBC-McMaster database	Self-identified shoulder pain	PSPI	SVM	Pain detection Estimation of pain intensity, categorized into four levels: PSPI 0, 1, 2, and ≥3	Pain detection Accuracy = 89.59% Pain intensity estimation Accuracy = 75%
Casti et al. (2021) [47]	UNBC-McMaster database	Self-identified shoulder pain	VAS	Linear discriminant analysis	Pain detection (VAS≥0) Pain intensity (VAS) estimation	Pain detection AUC = 0.87 Pain intensity estimation MAE = 2.44
Tavakolian et al. (2020) [48]	UNBC-McMaster database BioVid database (part A)	UNBC-McMaster database: self-identified shoulder pain BioVid database: heat-induced pain	UNBC-McMaster database: PSPI BioVid database: stimuli-based pain (5 levels)	CNNs	Estimation of pain intensity UNBC-McMaster: 16 pain levels BioVid: 5 pain levels	Training with BioVid and testing on UNBC-McMaster Self-supervised model: AUC = 69.2% Supervised model: AUC = 80.1% Training with UNBC-McMaster and testing on BioVid Self-supervised model: AUC = 65.5% Supervised model: AUC = 75.5%
Sikka et al. (2015) [49]	Pediatric patients from a tertiary care center	Postoperative pain	NRS	Logistic regression and linear regression models	Detection of clinically significant pain (NRS ≥ 4) Pain intensity (NRS) estimation	Clinically significant pain detection Baseline pain: AUC = 0.84 Transient pain: AUC = 0.91 Pain intensity estimation Baseline pain: r = 0.47; z = 4.4 * Transient pain: r = 0.47; z = 6.0 *

† Performance using leave-one-subject-out cross validation. * *p* < 0.0001. Abbreviations: UNBC-McMaster (UNBC-McMaster Shoulder Pain Archive), NRS (numeric rating scale), PSPI (Prkachin and Solomon Pain Intensity), VAS (visual analog scale), CERT (Computer Expression Recognition Toolbox), DML (Distance Metric Learning), AAMs (Active Appearance Models), CNN (Convolutional Neural Network), RNN (Recurrent Neural Network), LSTM (Long Short-Term Memory), SVM (Support Vector Machine), AUC (area under the curve), MSE (mean square error), MAE (mean absolute error).

## Data Availability

The databases generated for drafting this manuscript can be solicited from the author upon reasonable request.
